# Server pooling models for separate and bounded queues

**DOI:** 10.1016/j.heliyon.2024.e25344

**Published:** 2024-02-01

**Authors:** Hila Hindy Ling, Ran Etgar, Hillel Bar-Gera

**Affiliations:** a Ben-Gurion University of the Negev, Department of Industrial Engineering and Management, Israel; b Ruppin Academic Center, Department of Industrial Engineering and Management, Israel

**Keywords:** Pooling, QBD, Separate waiting queues, Container terminals, Arrival rates, Service rates

## Abstract

The focus of this paper is on queueing models with arrivals from several sources, where the number of waiting spaces for each arrival source is finite. The main distinction from prior studies is that in our models waiting spaces are separate. Within this context, we examine the benefit of pooling, regardless of whether arrival rates are equal or different. We present a Quasi-Birth-and-Death (QBD) model to address the general case, with simplified versions tailored to specific scenarios. One practical application of the proposed models can be found in the loading and unloading processes at container terminals.

We define a measure for stochasticity-related inefficiency, denoted relative interaction delay (RID), and analyze its behavior for the case of a single waiting space for each source. We show analytically that in the base model, the RID approximation is inversely proportional to the number of pooled queues. Numerical evaluations show an added benefit of pooling when arrival rates differ, observing a linear enhancement that is notably more pronounced.

## 1 Introduction

A common question in queuing theory is whether to pool the queues such that the servers can serve all the customers, or handle a system with separate queues such that each group of servers serves its own queue (e.g. Refs. [[1], [2], [3], [4], [5], [6], [7]]).

The basic model for server pooling, denoted M/M/C, assumes Markov arrival and service processes, C independent servers with identical service rates, and unlimited queue length [8,9]. This model consists of one queue with a given average arrival rate, denoted by λ.

The effect of queue pooling in this context can be analyzed by the classic comparison of C queueing systems of type M/M/1, to a single M/M/C queueing system. It is well known that such pooling reduces the variability of the workload between the servers, and thus improves all service performance measures, such as the average waiting times. In particular, Ding [10] showed that M/M/C pooling can achieve shorter task response time (average waiting time in the system) which improves cloud computing energy efficiency. He found that on average the M/M/20 task response time performed 55.7 % better than twenty M/M/1's when both cases servers are homogeneous with the same rates of service. Furthermore, M/M/20's average task response time was 68.7 % better than twenty M/M/1's (with heterogeneous servers and varying service rates). In general, Ding shows that the average waiting time in C systems of M/M/1 queue is always inferior to the average waiting time in M/M/C model when C > 1.

A prevalent variation of the M/M/C model presumes a limited capacity, N ≥ C, and is denoted as M/M/C/N. In this model, should customers arrive when N customers are already present in the system (either receiving service or waiting), they are unable to join the queue and must depart. An especially notable instance of these finite capacity models occurs when N=C , meaning there are no waiting spots available for arrivals during times when all servers are occupied. Such scenarios are referred to as pure "loss" models and are commonly utilized to assess service systems where having customers wait to commence service is deemed either impractical or highly undesirable [11].

In the case of N=C, there is no queue and therefore the waiting time is zero. The performance in this case can be measured by the effective arrival rate, $\lambda_{\text{eff}}$, or equivalently by its inverse, the average output time, $\text{AOT}=\frac{1}{\lambda_{\text{eff}}}$. Our focus is on the case of limited queues and thus the main performance measure would be the AOT.

Queueing models often assume similar arrival rates λ and similar service rates μ. Fibich et al. [12] Examined a queue comprising C heterogeneous servers, with distinct service rates denoted as $\mu_{1},...,\mu_{C}$. They argue that since certain conditions of differentiability and symmetry hold, an averaging principle applies, and therefore the effect of the magnitude of service rate variability on system performance should be quadratic. We are interested in the effect of pooling J queues while considering heterogeneous arrival rates $\lambda_{1},\lambda_{2},\ldots ,\lambda_{J}\text{.}$ This system satisfies the differentiability and symmetry conditions, and therefore the averaging principle theorem of Fibich et al. [12] holds as well.

In an M/M/C/N queue there are K=N−C waiting spaces. When pooling J queues of this type, it is natural to assume joint waiting spaces. Under this assumption, the pooled system can also be modeled by M/M/C/N, with $C_{\text{pool}}=J∙C$ servers, $K_{\text{pool}}=J∙K$ waiting spaces, and arrival rate of $\lambda_{\text{pool}}=J∙\lambda$. The M/M/C/N model can also handle the case of different arrival rates by setting $\lambda_{\text{pool}}={\sum \lambda }_{j}$.

The main goal of our research is to analyze the influence of pooling when the waiting spaces remain separate. We consider both the case with similar arrival rates and the case with different arrival rates. We define a measure for stochasticity-related inefficiency, denoted relative interaction delay (RID), and show that in the base model, the RID approximation is inversely proportional to the number of pooled queues.

While we consider different arrival rates, we focus on models with identical service rates. However, these models can be expanded to accommodate varying service rates per queue, as discussed and illustrated in Appendix A.

Separate waiting spaces may not be the general case, yet they can be found in various systems, such as container terminals, in the unloading and loading processes. Details about this test case are presented in section 2. To clarify the idea of separate waiting spaces, consider the following hypothetical scenario.

In the system, there are three distinct types of customers, and each customer type is allocated its own specific set of waiting spaces. If a customer of type I arrives at the system when all the servers and all type I waiting spaces are occupied, then, even if there are available waiting spaces for customers of type II or III, the customer will have to leave the system without getting a service. Servers can still work as one pool. When a server completes a previous service, it will be assigned to serve one of the waiting customers (if there are any, otherwise it will remain idle). The choice between customer types is made with the same probability without priorities. The choice between customers of the same type is based on first-come-first-served.

To summarize, the queuing models we will discuss here can be classified by several attributes: 1. With or without pool; 2. Finite/unlimited waiting spaces; 3. Joint/separate waiting spaces; and 4. Same/different arrival rates. The types of model needed for each of these cases are listed in Table 1.

**Table 1:** Queuing models for different scenarios.

Scenario	Pool	Waiting spaces	Number of waiting spaces	Arrival rates	Model
1	with/without	joint	infinite	same/different	M/M/C
2	with/without	joint	finite	same/different	M/M/C/N
3	with/without	separate	infinite	same/different	M/M/C
4	without	separate	finite	same/different	M/M/C/N
5	with	separate	0	same/different	M/M/C/N
6	with	separate	1	same	BD
7	with	separate	1	different	QBD
8	with	separate	2+	same	QBD
9	with	separate	2+	different	QBD

As discussed above, scenarios 1–5 have been considered in the literature. Specifically, scenario 5 describes a special case of pooling in which N=C, meaning there are no waiting spaces at all (K=0). Therefore, specifying whether waiting spaces are joint or separate is redundant. This case is listed in Table 1 only for the sake of completeness. Analysis of scenarios 6–9 is a key component of the contribution of the current paper, and the specific models needed for this purpose will be presented in section 3.

The rest of this paper is structured in the following manner: Section 2 presents a test case of a system with separate waiting spaces. In Section 3 we present the methodology of our research. Next, in Section 4, we present illustrative numeric results. Conclusions and recommendations for future research are provided in Section 5.

Practical application (test case)

The models discussed in this paper are suitable, among other things, when pooling at container terminals is considered. Container terminals play a crucial role as intermodal junctions within the worldwide transportation network. Effective management of container operations at these terminals is vital for minimizing transportation expenses and maintaining adherence to shipping timetables [13]. A primary goal in port operation, therefore, is to minimize the vessel turn-around time, i.e., the total makespan of the loading and unloading processes summed together. Loading and unloading involve quay cranes (QC), yard trucks (YT) and in some cases yard cranes (YC) and are thus multi-stage processes.

Carlo et al. [14] discussed the primary challenges involved in transport operations in container terminals and distinguished among three principal decision-making challenges: (1) choosing the appropriate type of vehicle, (2) deciding on the number of vehicles needed, and (3) planning the routes and dispatching the vehicles.

The type of yard truck vehicle can have a substantial effect on the analysis of other decisions. Yard trucks can be with or without lifting capabilities, automated or manually driven, and vary in other ways. If yard trucks do not have lifting capabilities, in the unloading process for example, when the QC picks up a container from a vessel, and there is no available YT, it must wait while holding the container and cannot continue bringing containers from the vessel. When automated lifting vehicles (ALVs) are used as YTs, they can pick containers from a buffer near the QC. Still, such buffers are typically limited in space area, and therefore, when the buffer is full, the crane can hold one extra container, but after that, new containers' arrival must stop. In either case, the number of waiting spaces near each crane is limited and separated from waiting spaces near other cranes, thus corresponding to scenarios 6–9 in Table 1. QC rates may be the same, or similar, but more often the rates vary due to cargo type (e.g. empty or full containers), QC operator skills (if not automated), etc. Therefore, the case of different arrival rates (scenarios 7 & 9) is more relevant in this context than the case of equal arrival rates (scenarios 6 & 8).

To clarify the connection between the queueing models discussed in this paper, and the joint operation of QCs and YTs, note that the operation of each tool can be described as a sequence of active cycles and idle intervals. Cycles for both QC and YTs begin at the same moment: during unloading when a container is released on a YT and during loading when a container is picked up from a YT. The end of every QC (YT) cycle is considered a QC (YT) event. Following a QC (YT) event, the QC (YT) may begin a new active cycle, or it may enter an idle interval.

In the model presented for scenarios 6 and 7 from Table 1, every QC event (i.e., the end of a QC cycle) is considered as a “birth” or as a new arrival to the system, and every YT event (i.e., the end of a YT cycle) is considered as a “death”. Following this interpretation, YTs are the servers, and YT “jobs” are the customers. Note that in the last part of the job in the unloading process and the first part of the job in the loading process, the YT does not carry a container, and thus, the container itself cannot be considered as the customer of the service.

It should be noted that other studies of stochastic analytic models for container terminals have used different interpretations of “customers” and “servers,” as summarized in Table 2. The above interpretation is relevant to our situation, as we concentrate on the interaction between quay cranes and yard trucks, whether they are pooled or not. In section 4 we present illustrative numeric results for the case of K=1. This case corresponds to regular YTs, where a QC can hold up to one job (and stay idle) if all the servers (YTs) are occupied.

**Table 2:** Interpretations of "customers” and “servers” in queueing models for port operations.

Citation	Customers	Servers
Dhingra et al. [15]	Automatic guided vehicles	Automated stacking cranes, QC, travel times
Zhang et al. [16]	Truck	Handling operations (virtual service process)
Easa [17]	Berths	Tugs
This paper	YT “jobs”	YTs

## 2 Methodology

As described in section 1, we consider a system with multiple queues, each queue belongs to a different type of entity and has its own waiting spaces. Consider J queues with a total of C servers working at an average rate of μ, where each queue has a different average arrival rate $\lambda_{1},\lambda_{2},\ldots ,\lambda_{J}$ and a separate waiting area that can hold up to K entities. We assume that inter-arrival times and service times follow exponential distributions, the system is memoryless and can be described as a Markov process. When each queue operates independently, their behavior can be described by the M/M/C/N model.

If all servers are pooled, but waiting areas remain separate, the model has two types of states. The first type of state describes the system with idle servers. Each state of this type is identified by the number of entities in the system, 0≤n<C, which is equal to the number of active servers. The transition rate from state n to state n+1 equals to the sum of all the arrival rates ($\sum_{j=1}^{J}\lambda_{j}$). The transition rate from state n to state n−1 depends on the number of active servers, and equals to n∙μ.

The second type of state describes the system when all servers are active. In this case, each state is identified by the number of entities in each queue, $0\leq b_{j}\leq K$. Hence, there are ${\left(K+1\right)}^{J}$ states of the second type, and $G=C+{\left(K+1\right)}^{J}$ possible states in total. The maximum quantity of entities present in the system is N=C+K∙J. This number is obtained when all the servers are active, and the waiting spaces of all queues are full.

**Fig. 1:**
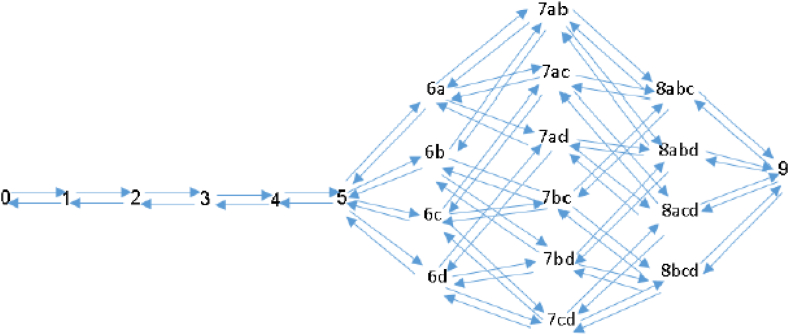
State diagram for four queues with different arrival rates, one waiting space in each queue and total of five servers (J=4,C=5,K=1).

To illustrate the state space, consider an example of four queues (a,b,c,d) and five servers working as a pool, when the number of waiting spaces in each queue is one (J=4,C=5,K=1). Fig. 1 presents the state diagram for this scenario. The first five states ('0′ to '4′) are the states with idle servers. The sixth state ('5′) is the state in which all the servers are active and all waiting spaces are empty. Consider next states with 6 entities in the system, meaning all the servers are active and there is one idle queue since its waiting space is full. The idle queue can be one of each of the four queues, so there are in fact four states in this situation - 6a,6b,6c,6d, when the number '6′ indicates the number of entities in the system and the letter indicates the idle queue. A 'birth' means a new arrival to the system, which can be only to one of the other queues. Following a “birth” there would be 7 entities in the system and 2 fully occupied queues (a and b, a and c, a and d, b and c, b and d, or c and d). Another ‘birth’ leads to 8 entities in the system, 5 of them in service and 3 in waiting spaces, such that the set of states in this case would be 8abc, 8abd, 8acd or 8bcd. The last state (‘9’) indicates a full system in which all waiting spaces are full and all the servers are busy, so there are 4 + 5 entities in the system.

In the general case, we define two related indices n and m such that each state can be represented by the pair (n,m):

n-number of entities in the system n=0,1,2,…,C+K∙J.

m-a combination of the waiting status of all areas $m=0,\ldots ,{\left(K+1\right)}^{J}-1$.

When n≤C (all waiting spaces are empty) then m gets the value of zero, and when n>C then m gets the value of $1,\ldots ,{\left(K+1\right)}^{J}-1$. Let $b_{j}\left(m\right)$ be the jth digit of m, when represented in base (K+1), i.e. $m=\sum_{j=1}^{J}b_{j}\left(m\right)∙{\left(K+1\right)}^{j-1}\text{.}$ We consider $b_{j}\left(m\right)$ as describing the number of occupied waiting spaces in queue j. For example, state 6b in Fig. 1 would be described as $\left({6,2}_{\text{dec}}=0010_{\text{bin}}\right)$ meaning that there are 6 entities in the system and there is one entity in the second queue. This entity denotes a full queue because k = 1. In this case the digits are being represented by binary representation – '0′ for empty queue and '1′ for full queue. Note that when m>0 $n=C+\sum_{j=1}^{J}b_{j}\left(m\right)$.

A birth is represented by a single-digit increase by 1, while all the other digits remain unchanged, and a death is represented by a single-digit decrease by 1, while all the other digits remain unchanged.

We define w_j_ as an indicator describing whether waiting area j is active (not empty):

(1) $$w_{j}\left(b_{j}\right)=\left\{ \begin{array}{c} 0\;b_{j}=0\\ 1\;\text{else}\; \end{array}\right.$$

From eq. (1), W(m) can be defined as the number of active waiting areas:

(2) $$W\left(m\right)=\sum_{j=1}^{J}w_{j}\left(b_{j}\left(m\right)\right)$$

when m > 0 all servers are busy. From eq. (2), when a server becomes available, the transition will be to a state with

(3) $$\hat{m}\in S^{-}\left(m\right)=\{\hat{m}|\exists j\text{,}b_{j}\left(\hat{m}\right)=b_{j}\left(m\right)-1\geq 0;\;b_{j^{\prime }}\left(\hat{m}\right)=b_{j^{\prime }}\left(m\right)\;\forall j^{\prime }\neq j\}$$

There are $W\left(m\right)=\left|\;S^{-}\left(m\right)\right|$ such possible transitions. Subsequently, we will also use a corresponding set of non-empty waiting areas $J^{-}\left(m\right)=\{j|b_{j}\left(m\right)>0\}$.

The sum of these transition rates is the rate that a server becomes available, i.e. C∙μ. According to our assumption, the probability of choosing each one of these transitions is $\frac{1}{W\left(m\right)}$. Therefore, the probability of the transition from state (n,m) to state $\left(n-1,\hat{m}\right)$, where $\hat{m}\in S^{-}\left(m\right)\text{,}$ equals to $C∙\mu ∙\frac{1}{W\left(m\right)}$.

The arrival rate of entity of type j is $\lambda_{j}\;\left(j=1,2,..,J\right)$, and this is the transition rate from state (n,m) to state $\left(n+1,\hat{m}\right)$ where

(4) $$\hat{m}\in S^{+}\left(m\right)=\{\hat{m}|b_{j}\left(\hat{m}\right)=b_{j}\left(m\right)+1\leq k;\;b_{j^{\prime }}\left(\hat{m}\right)=b_{j^{\prime }}\left(m\right)\;\forall j^{\prime }\neq j\}$$

Subsequently, we will also use a corresponding set of non-full waiting areas:

$$J^{+}\left(m\right)=\{j|b_{j}\left(m\right)<K\}\text{.}$$

The transition matrix of the system's states is represented in Table 3.

**Table 3:** State transition rates of pooling queues with different arrival rates (all other transition rates are zero).

From state	To state	Conditions	Rate
n,0	n+1,0	0≤n<C	$\sum_{j=1}^{J}\lambda_{j}$
n,0	n−1,0	0<n≤C	nμ
n,m	$n+1,\hat{m}$	$0\leq m<{\left(K+1\right)}^{J}$; 0<$\hat{m}\leq {\left(K+1\right)}^{J}$$j\in J^{+}\left(m\right)\text{;}$$b_{j}\left(\hat{m}\right)=b_{j}\left(m\right)+1\leq$ K; $b_{j^{\prime }}\left(\hat{m}\right)=b_{j^{\prime }}\left(m\right)\;\forall j^{\prime }\neq j$	$\lambda_{j}$
n,m	$n-1,\hat{m}$	$0<m\leq {\left(K+1\right)}^{J}$; $0\leq \hat{m}<{\left(K+1\right)}^{J}$$j\in J^{-}\left(m\right);b_{j}\left(\hat{m}\right)=b_{j}\left(m\right)-1\geq 0;\;b_{j^{\prime }}\left(\hat{m}\right)=b_{j^{\prime }}\left(m\right)\;\forall j^{\prime }\neq j$	$\frac{C\mu }{W\left(m\right)}$

The steady-state probability of state (n,m) is denoted by $P_{n,m}$. Denote by ***P*** a diagonal matrix of dimension G×G, with all state probabilities. Denote by **STR** the square matrix of state transition rates, as given in Table 3. Let $J={\left(1,\ldots ,1\right)}^{t}$ be a vector of ones of the same dimension. Having eqs. (3), (4)), we can calculate $P_{n,m}$ using the equations:

(5) $$\left({\text{STR}}^{t}\cdot P-P\cdot \text{STR}\right)\cdot J=0;\;\sum_{n,m}P_{n,m}=1$$

Eq. (5) enables the creation of Table 3.

Once we have the steady-state probabilities, based on eqs. (6), (7), (8), $\lambda_{\text{eff}}$ can be calculated, which is the actual arrival rate, while considering all the states in which a birth occurs, meaning a new entity arrives in the system.

(6) $$\lambda_{\text{eff}1}=\sum_{j=1}^{J}\lambda_{j}\cdot \sum_{n=0}^{C-1}P_{n,0}$$

(7) $$\lambda_{\text{eff}2}=\sum_{m=0}^{{\left(K+1\right)}^{J}-1}\sum_{j\in J^{+}\left(m\right)}\lambda_{j}∙P_{n\left(m\right),m}$$

(8) $$\lambda_{\text{eff}}=\lambda_{\text{eff}1}+\lambda_{\text{eff}2}$$

We are considering systems where the focus is queue output, and therefore, a key performance measure is the average output time (AOT) per entity per queue. It can be computed by dividing the number of queues by $\lambda_{\text{eff}}$ ,as depicted in eq. (9).

(9) $$\text{AOT}=\frac{J}{\lambda_{\text{eff}}}$$

From here on we shall assume that each queue has C_1_ servers, and hence there are $C=J∙C_{1}$ servers in total. We assumed that each queue may have a different arrival rate, $\lambda_{j}$ , and denote the average arriving rate by $\lambda =\frac{\sum_{j=1}^{J}\lambda_{j}}{J}$. Considering that $\lambda_{\text{eff}}\leq \sum_{j=1}^{J}\lambda_{j}=J∙\lambda$ and $\lambda_{\text{eff}}\leq C∙\mu$, we get a lower bound for AOT (eq. (10)):

(10) $$\text{AOT}\geq \text{LB}=\max\left\{\frac{J}{J∙\lambda },\frac{J}{C∙\mu }\right\}=\max\left\{\frac{1}{\lambda },\frac{1}{C_{1}∙\mu }\right\}$$

We define θ as the ratio of arrival rates to service rates, thereby capturing the demand-to-supply ratio (eq. (11)):

(11) $$\theta =\frac{J∙\lambda }{J∙C_{1}∙\mu }=\frac{\lambda }{C_{1}∙\mu }$$

Therefore, the lower bound (eq. (12)) is:

(12) $$\text{LB}=\left\{ \begin{array}{c} \frac{1}{C_{1}∙\mu }\;\theta \geq 1\\ \frac{1}{\lambda }\;\theta \leq 1 \end{array}\right.$$

If the system were deterministic, the lower bound (12) would prevail. In particular, when θ=1, $\lambda =C_{1}∙\mu$, arrivals and departures are synchronized and there are no delays in the system. When variances exist, AOT may be higher than LB. These additional delays are due to the stochastic interaction between the arrival process and the service process. Our focus is on the gap between AOT and LB. To capture this gap, we define the relative interaction delay (RID) as the relative difference between the AOT and its LB, as depicted in eqs. (13), (14)):

(13) $$\text{RID}=\frac{\text{AOT}}{\text{LB}}-1$$

(14) $$\text{RID}=\left\{ \begin{array}{c} \frac{\frac{J}{\lambda_{\text{eff}}}}{\frac{1}{C_{1}∙\mu }}-1\;\theta \geq 1\;\\ \frac{\frac{J}{\lambda_{\text{eff}}}}{\frac{1}{\lambda }}-1\;\theta \leq 1 \end{array}\right.=\left\{ \begin{array}{c} J∙\frac{C_{1}∙\mu }{\lambda_{\text{eff}}}-1\;\theta \geq 1\;\\ J∙\frac{\lambda }{\lambda_{\text{eff}}}-1\;\theta \leq 1 \end{array}\right.=\left\{ \begin{array}{c} \frac{C∙\mu }{\lambda_{\text{eff}}}-1\;\theta \geq 1\;\\ \frac{J∙\lambda }{\lambda_{\text{eff}}}-1\;\theta \leq 1 \end{array}\right.$$

Pooling does not change the available resources, and therefore does not change LB. The change in RID is in this case the main contribution of pooling. In the next section we illustrate the influence of various factors on RID, and use these results to gain insights into the benefits of pooling.

The model presented here for general K corresponds to scenario 9 in Table 1. Scenario 8 is a special case with equal arrival rates, but the same model is needed. Scenario 7 is also a special case, with K=1. It requires a QBD model of a similar structure, although some minor technical simplifications can be implemented. Scenario 6 can be treated as a special case of scenario 9, but since arrival rates are the same and K=1, the following BD model is sufficient. In this scenario, as long as there are fewer entities than active servers, a new arrival, or birth, can occur, with the rate of J∙λ. When n, which is the number of entities in the system, is bigger than the total number of servers, C, then the entities will have to wait (at most one in each queue), and new arrivals will occur only in queues with available waiting space. Thus, the arrival rate will be (N−n)∙λ. A full system means that all the servers are busy and all the spaces of all types of entities are occupied, so there is a maximum of N=J+C entities in the system.

**Fig. 2:**
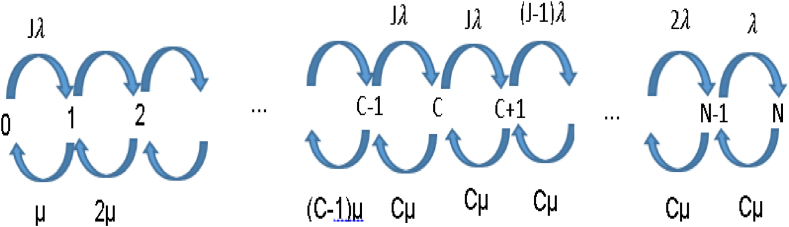
Transition diagram for scenario 6: pool with similar arrival rates and separate waiting spaces.

Fig. 2 and Table 4 present the Markov chain and the system transition rates matrix for this model.

**Table 4:** State transition rates of BD model for pool with similar arrival rates and separate waiting spaces.

From state	To state	Condition	Rate
N	n+1	0≤n<C	Jλ
N	n+1	C≤n≤N−1	(N−n)λ
N	n−1	0<n≤C	nμ
N	n−1	C<n≤N	Cμ

$\lambda_{\text{eff}}$ and AOT are calculated as follows:

$$\lambda_{\text{eff}}=\sum_{n=0}^{C-1}J\lambda ∙P\left(n\right)+\sum_{n=C}^{N-1}\left(N-n\right)\lambda ∙P\left(n\right)$$

$$\text{AOT}=\frac{J}{\lambda_{\text{eff}}}$$

This model may seem similar to the standard M/M/C/N model, but it is not fully equivalent, because arrival rates are state-dependent. The ability to represent scenario 6 by a birth-and-death model, which is simpler to analyze than the more general QBD model, allows for a more rigorous analysis of its properties. Specifically, the effectiveness of pooling can be proven to hold in general. The details of the proof are presented in Appendix B.

## 3 Illustrative numeric results

To better understand the effects that various factors can have on pooling queues it is useful to consider specific numerical examples. We start the evaluation with the reference case, where the queues are not pooled and the arrival rates for the queues are similar. Next, we present the model's results when the queues are pooled, both when the arrival rates are similar, and when they differ. At the end of this section, we present a comparison between the pooling and non-pooling policies. The analysis focuses on the case in which each queue can hold up to 1 entity at most (K=1).

### 3.1 Non-pooling with similar arrival rates

**Fig. 3:**
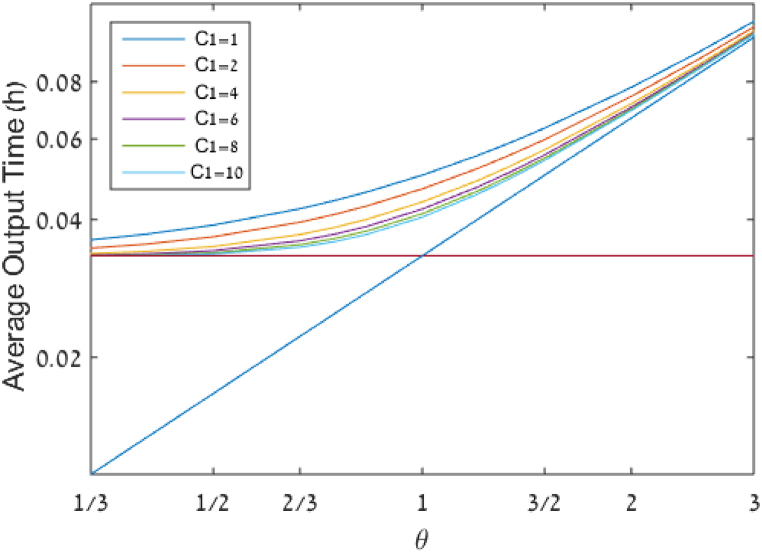
Average output time (AOT) per entity as a function of demand-to-supply ratio (θ) and the number of servers per queue ($C_{1}$). The arrival rate is λ=30 customers/hour.

Fig. 3 shows how AOT changes as a function of θ in the range between $\frac{1}{3}$ to 3 for various options in terms of the number of servers per queue: $C_{1}=1,2,4,6,8,10$. For the sake of this illustration, the average arrival rate (λ) is 30 units per hour. For every combination of parameters, the value of $\text{AOT}\left(\lambda ,\mu ,C_{1}\right)$ as defined in section 1 is computed by using the equations presented there. Note that when λ and $C_{1}$ are given, θ can be determined from μ and vice versa.

As observed from Fig. 3, for a given number of servers per queue $C_{1}$, AOT increases as a function of θ, exhibiting a progressively steeper slope. Two lower bounds are observed (explained in section 3): The first one is dictated by the arrival rate, as $\text{AOT}\geq \frac{1}{\lambda }$ and is represented in Fig. 3 by the solid horizontal line. The second one is dictated by the service rate, $\text{AOT}\geq \frac{1}{C_{1}∙\mu }=\frac{\theta }{\lambda }$ and represented in Fig. 3 by the solid diagonal line, which is linear in θ, and crosses the first lower bound when θ=1. When θ≪1, i.e., towards the left side of the graph, performance is dominated by the arrival rate. When θ≫1, i.e., towards the right side of the graph, performance is dominated by the service rate. In all cases, the more limiting bound governs.

**Fig. 4:**
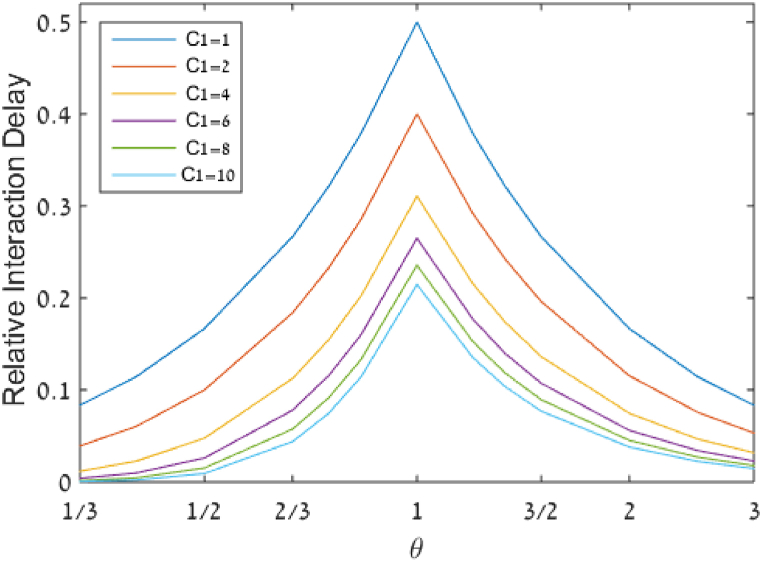
Relative interaction delay (RID) as a function of demand-to-supply ratio (θ) and the number of servers per queue $\left(C_{1}\right)$.

As indicated previously, pooling cannot affect the lower bound, but it can affect AOT. Therefore, we focus our attention on the gap between AOT and the lower bound, which is represented by the RID, as defined in section 3. Fig. 4 describes the RID as a function of θ and $C_{1}$.

Fig. 4 shows clearly that for a given number of servers, the maximum value of RID is obtained when θ=1 and that RID values decrease as θ deviates further from 1. Fig. 4 also better depicts the quantitative effect of the number of servers on the RID for any specific value of θ, for example, the reduction in RID from 50 % to 40 % and 23.6 % when θ=1 as the number of servers per queue increases from 1 to 2, and 8, respectively.

### 3.2 Pooling with similar arrival rates

312 scenarios were examined for the case that the queues are pooled and the arrival rates to the queues are the same. In addition to the parameters we used in 4.1, we picked 4 values for the number of pooled queues, J: 1, 2, 4 and 8. Note that J=1 describes the case in which the queues are not pooled and is used as a reference for the other scenarios. Also, note that the number of servers per queue, $C_{1}$, dictates the total number of servers in the system. For example, when there are 6 servers per queue $\left(C_{1}=6\right)$ in the case that there is a pool of 4 queues (J=4), the total number of servers is C=24.

**Fig. 5:**
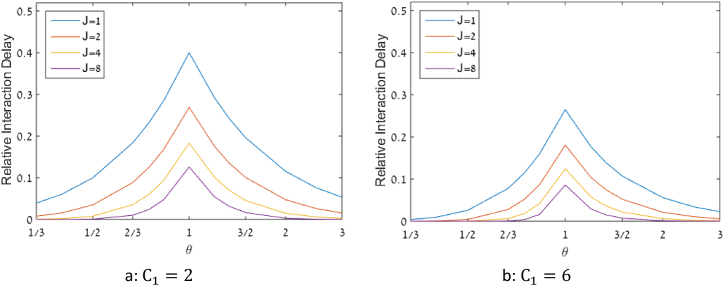
RID in different scenarios for pooling with similar arrival rates.

Examples for the RID values obtained in the examined scenarios are shown in Fig. 5.

From Fig. 5 it can be observed that the general pattern of RID as a function of θ, as discussed above, is similar in all the curves, pooled or not pooled. Additional conclusions from Fig. 5 are: i) For a given $C_{1}$, RID is a monotonically decreasing function of the number of pooled queues, thus demonstrating the benefit of pooling. For example, in the case of $C_{1}=6$, pooling 8 queues reduces RID from 0.265 to 0.086. ii) The largest improvement in RID values is obtained in the transition from the non-pooling option (J=1) to pooling of two queues (J=2). iii) The pooling advantage is more significant when $C_{1}$ is low. For example, when $C_{1}=2$, pooling 8 queues reduces RID from 0.4 to 0.126.

### 3.3 Pooling with different arrival rates

When each queue has a different arrival rate, we analyze the effect of the variance between the arrival rates on system metrics. We selected arrival rates in three dispersion ranges, such that the average of each range is 30 units per hour, and the scattering within the range is in even intervals. For example, the values of λ in the range between 20 and 40 when two queues are being pooled are 20 and 40, while in a pool of four queues, the values of λ are 20, 26.67, 33.33 and 40. Note that due to the small number of elements, the manner of dispersion of two items cannot be equivalent to the manner of dispersion of four or eight items. In particular, enlarging the pool lowers the variance between the values of λ. The selected ranges for rates are: Range=10,20,40 (20≤λ≤40,25≤λ≤35,10≤λ≤50). The results obtained for these three ranges were compared to the case in which the arrival rates are the same and equal to the average rate of the other ranges (λ=30,Range=0).

**Fig. 6:**
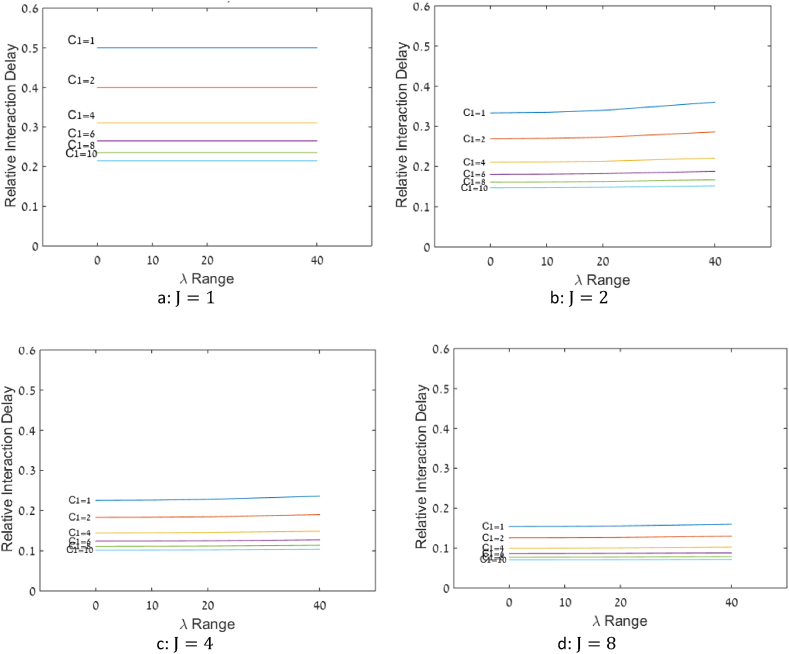
RID as a function of different arrival rates and different number of servers, θ=1. 6a: J=1. 6b: J=2. 6c: J=4. 6d: J=8.

72 scenarios were examined for the case that the queues are pooled and the arrival rates of the queues are different. In all of them the supply to demand ratio, θ, is one. Fig. 6 presents the values of RID obtained in different scenarios in each dispersion range.

Fig. 6 shows that in all of the scenarios, the values of RID increase as the dispersion between arrival rates increases. For each J, increasing $C_{1}$ lowers the influence of the dispersion between arrival rates on RID values. For each $C_{1}$, RID decreases as J increases. In all of the scenarios RID decreases as $C_{1}$ increases and the most significant improvement in RID is in the transition from $C_{1}=1$ to $C_{1}=$ 2.

As stated in section 1, based on Fibich et al. [12], since the conditions of differentiability and symmetry hold, the effect of λ -range on RID should be quadratic. Indeed, in our results the correlations between the range squared and the RID were all above 99.6 %. Due to the quadratic relationship, it is sufficient to consider only one range, as representative of all other ranges.

### 3.4 Pooling Vs. non-pooling policies with different arrival rates

**Fig. 7:**
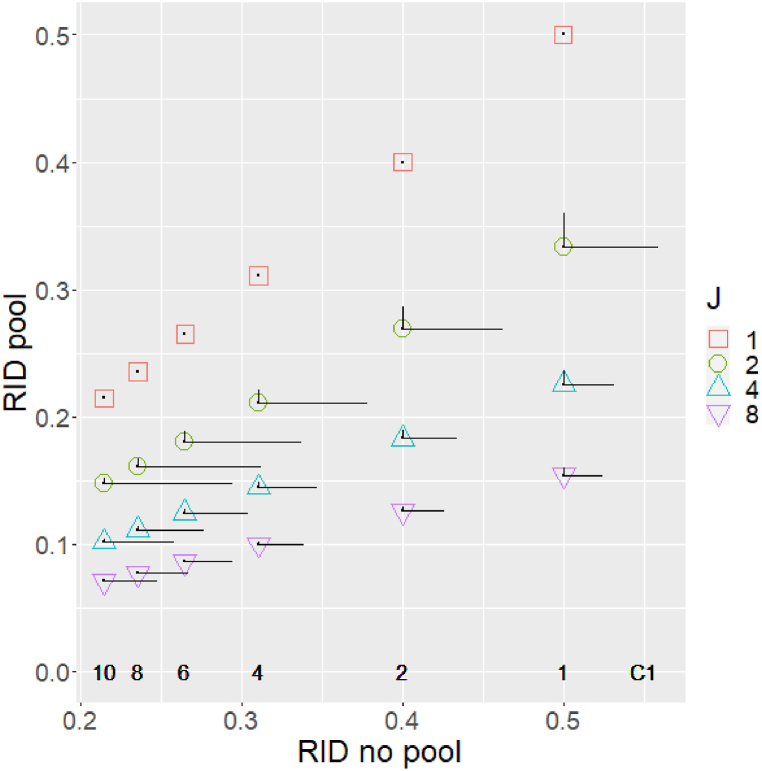
RID in different scenarios.

Fig. 7 summarizes the main results differently, to further clarify the impacts of pooling. The coordinates of each point in the graph present the RID value obtained in the non-pooling policy (the horizontal axis) and in the pooling policy (the vertical axis) when all the arrival rates are the same. Each point represents a different combination of the number of queues (symbol and color) and the number of servers per queue ($C_{1}$). Note that the non-pooling RID (horizontal axis) is not influenced by the number of queues, and therefore each “column” of points can be associated with a specific value of $C_{1}$, which is indicated at the bottom of the figure. An explanation for the nearly linear trend in the points of each sequence in this curve is provided in Appendix B.

The impact of variability in arrival rates is shown by “error bars”, i.e. the horizontal and vertical lines stemming from each point (for J>1). The horizontal line from each point to the right represents the RID increase for Range=20 in the non-pooling policy. The vertical line from each point upwards represents the RID increase for Range=40 in the pooling policy.

For example, the point (0.5,0.33) in the graph means that for θ=1, Range=0, $C_{1}=1$ and J=2 the RID is 0.5 in the non-pooling policy and 0.333 in the pooling policy, i.e. a ratio of 1.5. Increasing the range to 20 raises the RID value to 0.56 under the non-pooling policy, and increasing the range to 40 raises the RID value to 0.36 under the pooling policy.

**Fig. 8:**
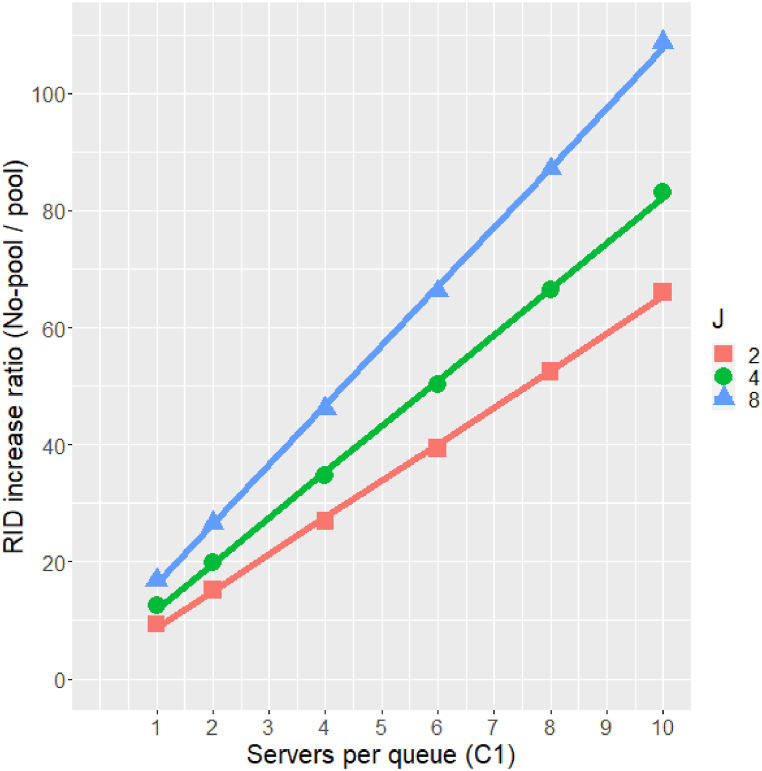
RID increase ratio as a function of $C_{1}$ and J, Range=20, θ=1.

Note: given the quadratic impact of range on RID increase (as discussed above), modifying vertical error bars to reflect Range=20 would make them 4 times smaller, and thus barely observable. Similarly, modifying horizontal error bars to reflect Range=40 would make them 4 times larger, thus clattering the figure and making it illegible. This choice of different ranges is necessary for figure clarity, but it does not permit a proper direct comparison of the impact of arrival rate variability between the pooling and non-pooling cases. Such a comparison will be presented later using Fig. 8.

Fig. 7 clearly shows that without pooling the impact of variability in arrival rates (Range=20) is quite substantial compared to the RID without variability. For example, in the case of J=2, $C_{1}=10$, the RID of 0.214 when arrival rates are the same, increases by 0.08–0.294 when the Range = 20. With pooling the relative impact of arrival rate variability is substantially less significant, even for Range=40. For example, in the case of J=2, $C_{1}=1$, the RID of 0.333 when arrival rates are the same, increases by 0.026–0.36 when the Range=40.

In addition to the impact of arrival rate variability, Fig. 7 also summarizes the impact of pooling when arrival rates are the same. The points associated with a given number of queues follow straight lines, reflecting constant ratios of pooling to non-pooling RID of 1, 0.66, 0.45 and 0.31 for J = 1, 2, 4, and 8 queues respectively.

Finally, we point out that the enlargement in RID values due to the differences in the arrival rates depends on the number of queues, both in the pooling and in the non-pooling policies. For the same value of $C_{1}$, both the horizontal line and the vertical line are longer as J decreases (except for one queue, obviously). We assume that the reason for this trend under the non-pooling policy is due to the differences between the distributions of arrival rates, and especially due to the differences between the variances (under the same range) as a function of the number of queues.

As mentioned above, Fig. 7 does not permit a proper direct comparison of the impact of arrival rate variability between the pooling and non-pooling cases. To enable such comparison, Fig. 8 describes the RID increase ratio at Range=20 as a function of the number of servers per queue. For example, for J=2 and $C_{1}=1$ the RID value in the non-pooling policy increases from 0.5 to 0.559 and in the pooling policy it increases from 0.333 to 0.339, so the RID increase ratio is $\frac{0.559-0.5}{0.339-0.333}=9.83$. This value is the lowest ratio that we obtained by changing policies from the non-pooling policy to the pooling policy. The pooling benefit increases with $C_{1}$ and with J. For example, for eight queues and ten servers the RID increase ratio is 108.68. As Fig. 8 shows, for a given number of queues, the increase ratio is linear in the number of servers per queue ($R^{2}=0.9997)$, according to the following calculations:

$${\text{RIDIncreasRatio}\left(J=2\right)=2.469+6.278C}_{1}$$

$${\text{RIDIncreasRatio}\left(J=4\right)=4.083+7.819C}_{1}$$

$${\text{RIDIncreasRatio}\left(J=8\right)=6.089+10.17C}_{1}$$

While we do not have a theoretical explanation for these linear trends, the match is quite striking. The main conclusion from Fig. 8 is that the impact of the variation between the arrival rates is much larger when the work is under the non-pooling policy, compared to the results with the pooling policy. Put in another way, when arrival rates are different the benefit of pooling is substantially higher.

## 4 Conclusions and future work

In this paper we examined the contribution of pooling queues with finite waiting spaces while maintaining separated waiting areas for each queue. We compared the non-pooling policy to the pooling policy both when the arrival rates are similar and when they differ. We mainly focused on a special case that involved only a single waiting space in each queue. This type of system finds practical application in container terminals, particularly in the processes of unloading and loading containers.

The main operational measure we examined is the relative interaction delay (RID), which is defined as the gap between the average output time and its lower bound. We found that when the arrival rates are similar, for a given number of servers, the RID is a monotonically decreasing function of the number of pooled queues, the largest improvement in RID values is obtained in the transition from the non-pooling option to pooling of two queues and the pooling advantage is more significant when the number of servers is low.

When there are different arrival rates, the values of RID increase as the dispersion between arrival rates increases. For each number of queues, increasing the number of servers per queue lowers the influence of the dispersion between arrival rates on RID values. For each number of servers per queue, RID decreases as the number of queues increases. RID decreases as the number of servers per queue increases and the most significant improvement in RID is in the transition from a single server to two servers per queue.

By comparing between the two options, we found that without pooling the impact of variability in arrival rates is quite substantial compared to the RID without variability. In addition, the enlargement in RID values due to the differences in the arrival rates depends on the number of queues, both in the pooling and in the non-pooling policies. The pooling benefit increases with the number of servers per queue and with the number of queues.

While this paper offers valuable practical insights, its contribution could be further extended through additional research, which would shed light on a wider range of scenarios. In the context of this paper, we assumed that servers select active queues randomly, without favoring any particular entity type. An alternative avenue for future research could involve exploring different assumptions, where servers consider various characteristics – such as importance, size, and more – when choosing which queue to serve.

Another promising direction for expanding this research involves relaxing the assumption of exponentially distributed service times. Although this assumption has its advantages, it does not cover all possible cases. Therefore, it is important to investigate how different service time distributions might impact the conclusions presented here.

## CRediT authorship contribution statement

**Hila Hindy Ling:** Conceptualization, Formal analysis, Methodology, Software, Writing – original draft, Writing – review & editing. **Ran Etgar:** Methodology, Supervision, Writing – review & editing. **Hillel Bar-Gera:** Conceptualization, Formal analysis, Methodology, Supervision, Writing – original draft, Writing – review & editing.

## Declaration of competing interest

The authors declare that they have no known competing financial interests or personal relationships that could have appeared to influence the work reported in this paper.

## Figures and Tables

**Fig. 9:**
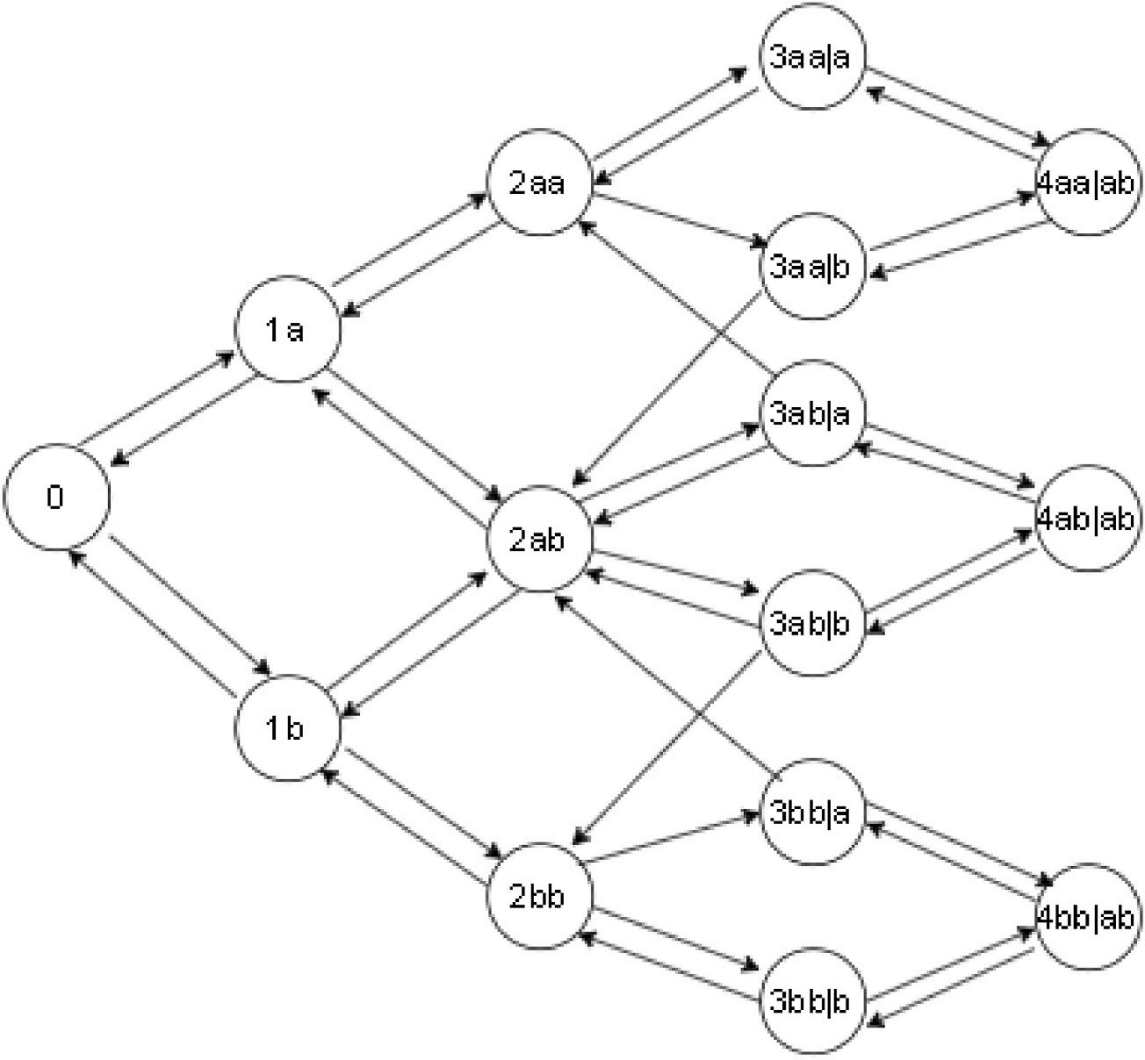
States diagram for 2 classes of customers, 2 servers and maximal capacity of 1 in each queue

**Table 5:** Transition matrix for 2 classes of customers, 2 servers and maximal capacity of 1 in each queue.

Rate	From state- > To state
$\lambda_{a}$	0-1a; 1a-2aa; 2aa-3aaa; 1b-2ab; 2ab-3aba; 2bb-3bba; 3aab-4aaab; 3abb-4abab; 3bbb-4bbab
$\lambda_{b}$	0–1b; 1b-2bb; 2bb-3bbb; 1a-2ab; 2ab-3abb; 2aa-3aab; 3bba-4bbab; 3aba-4abab; 3aaa-aaaab
$\mu_{a}$	1a-0; 2ab-1b; 3abb-2bb; 3aba-2ab; 4aaab-3aaa; 4aaab-3aab
$\mu_{b}$	1b-0; 2ab-1a; 3abb-2aa; 3abb-2ab; 4bbab-3bbb; 4bbab-3bba
${2\mu }_{a}$	2aa-1a; 3aaa-2aa; 3aab-2ab
2 $\mu_{b}$	2bb-1b; 3bbb-2bb; 3bba-2ab
$\frac{\mu_{a}+\mu_{b}}{2}$	4abab-3aba; 4abab-3abb

**Fig. 10:**
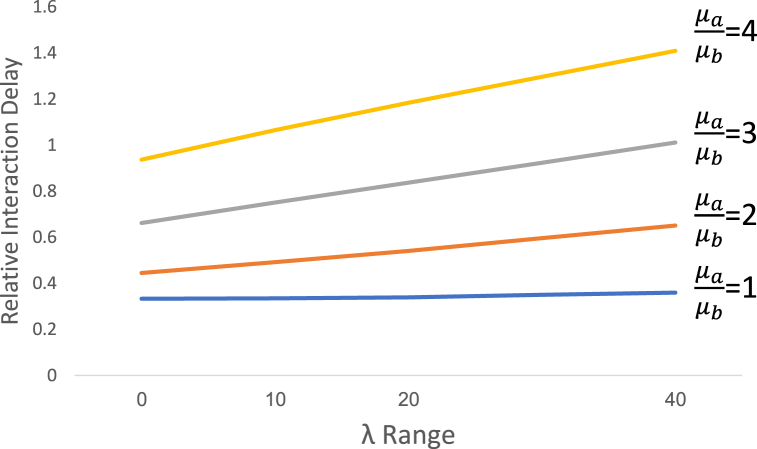
RID as a function of different arrival rates and different ratio of service rates, θ=1, J = 2, C = 2, K = 1.

## Appendix A Accommodating different service rates

In the models presented throughout this paper, we assumed that the distribution of service time is the same for all queues and particularly the average service rate. In practice, arrival times and service times may be affected by many factors. Some of the factors are related to the specific item. For example, in the context of container terminals, a specific container may be empty or full. Item specific factors may contribute to the distribution of arrival and service times within each queue, and they may also cause differences between queues. In the context of container terminals, item-specific attributes may affect the operations of quay cranes (i.e., arrival rates in our model) or yard trucks (i.e., service rates in our model). Other factors, like the experience of a human crane operator, or the maintenance level of the quay crane, may influence the quay crane process and hence lead to different arrival rates, even if the properties of items within each queue are similar. Factors associated with the quay crane and its operator are not likely to affect the yard trucks, i.e., the service rates of different queues. Therefore, at least in the context of container terminals, differences between the average service rates (yard truck operations) of separate queues (different cranes) are often sufficiently modest, so it is appropriate to rely on the models we analyzed so far. In other cases, whether related to container terminals or not, an extension of the proposed models to address different service rates may be useful.

The main implication of the extension for different service rates is a substantial increase in the number of states in the QBD model. Even if the capacity of each queue is limited to one, the total number of states is:

$$\sum_{n=0}^{C}\left(\begin{array}{c} J+n-1\\ n \end{array}\right)+\sum_{i=1}^{J}\left(\begin{array}{c} J+C-1\\ C \end{array}\right)∙\left(\begin{array}{c} J\\ i \end{array}\right)\text{,}$$

Where the first sum considers states with n≤C entities in the system, and the second sum considers states with i≤J queues where the capacity of one is utilized.

To further illustrate the implications of such an extension, the remainder of this appendix focuses on the case of two queues with one server in each queue ($C_{1}=1$), and a maximum queue capacity of one in each queue (K = 1). Similar to other models in this paper, we assume Poisson processes, with arrival rates of $\lambda_{a}$ and $\lambda_{b}$, and service rates of $\mu_{a}$ and $\mu_{b}$. Possible state transitions are shown in Fig. 9, and their probabilities are listed in Table 5. RID can be derived following similar procedures to equations (5), (6), (7), (8), (9), (10), (11), (12), (13), (14), with a few minor modifications.

The main differences in the derivations are.

1. The lower bound is:

(15) $$LB=\min\left(\frac{2}{\lambda_{a}+\lambda_{b}},\frac{2}{\mu_{a}+\mu_{b}}\right)$$

2. the potential state-dependent arrival rates implied by Table 5 are:

(16) $$\Lambda_{s}=\left\{ \begin{array}{cc} 0 & s\in \left\{4aa\left|ab;4ab\right|ab;4bb|ab\right\}\\ \lambda_{a} & s\in \left\{3aa|b;\;3ab\left|b;\;3bb\right|b\right\}\\ \lambda_{b} & s\in \left\{3aa|a;\;3ab\left|a;\;3bb\right|a\right\}\\ \lambda_{a}+\lambda_{b} & otherwise \end{array}\right.$$

3. the effective arrival rate is:

(17) $$\lambda_{eff}=\sum_{s\in S}\Lambda_{s}\cdot P\left(s\right)$$

Where S is the set of all possible states.

16 scenarios were examined for this case of two queues, each with its own arrival rate and service rate. The selected ranges for arrival rates were: Range=10,20,40 $\left({\;\lambda }_{a}=20,{\;\lambda }_{b}=40;\;\lambda_{a}=25,{\;\lambda }_{b}=35;\;\lambda_{a}=10,\lambda_{b}=50\right)$. The results obtained for these three ranges were compared to the case in which the arrival rates are the same and equal to the average rate of the other ranges $\left(\lambda_{a}=\lambda_{b}=30,\text{Range}=0\right)$. Four service rates' ratios between the two queues were selected as: 1:1, 2:1, 3:1 and 4:1. Their values were a result of the calculation of the supply to demand ratio, θ, which was equal to one in all the scenarios. The RID values obtained in the 16 scenarios are presented in Fig. 10.

As observed in Fig. 10, the simple instance where the ratio is 1 (implying identical service rates) aligns with the outcomes illustrated in Fig. 6. The greater the ratio (signifying a disparity in service rates), the more pronounced the departure of the Average Output Time (AOT) from the lower bound. Similarly, heightened divergence from the lower bound occurs as the discrepancy between arrival rates increases.

## Appendix B Formal analysis of pooling efficiency

One of the key findings that is shown consistently in the numerical analysis is that pooling enhances efficiency. The purpose of this appendix is to provide a formal mathematical analysis of this finding under one simplifying assumption, namely that the capacity in each queue is one (K = 1), i.e. that we can use the BD model of scenario 6. Recall that in scenario 6 the number of states is C+J, where $C=C_{1}J$.

Using node balance equations:

(18) $$P_{C-i-1}\cdot \lambda \cdot J=P_{C-i}\cdot \mu \cdot \left(C-i\right)\;\Rightarrow \;P_{C-i-1}=P_{C-i}\theta^{-1}\frac{C-i}{C}\;\forall 1\leq i\leq C$$

(19) $$P_{C+j}\cdot \lambda \cdot \left(J-j\right)=P_{C+j+1}\cdot \mu \cdot C\;\Rightarrow \;P_{C+j}\theta \frac{J-j}{J}=P_{C+j+1}\;\forall 1\leq j\leq J$$

We find the connection between each state probability and the probability of the “critical” state where all servers are busy but there is still no item in the queue:

(20) $$P_{C-i}=P_{C}\theta^{-i}\frac{C!}{C^{i}\left(C-i\right)!}\;\forall 0\leq i\leq C-1$$

(21) $$P_{C+j}=P_{C}\theta^{j}\frac{J!}{J^{j}\left(J-j\right)!}\;\forall 0\leq j\leq J-1$$

We can rewrite (20–21) as $P_{C-i}=P_{C}\theta^{-i}f_{C,i}$ and $P_{C+j}=P_{C}\theta^{j}f_{J,j}$, by denoting:

(22) $$f_{N,k}=\frac{N!}{N^{k}\left(N-k\right)!}$$

Lemma 1 the sequence $f_{N,k}$ for a given 1≤k≤N, is increasing with N, and $0<f_{N,k}<1$. Proof: for 1≤k≤N−1 we can rewrite the term as $f_{N,k}=\frac{N!}{N^{k}\left(N-k\right)!}=\prod_{j=1}^{k-1}\frac{N-j}{N}$. For a given 1≤j≤N−1, each term $\frac{N-j}{N}$ is increasing with N, and $0<\frac{N-j}{N}<1$. Therefore, these properties are maintained in the product $f_{N,k}$. For k=N we get $f_{N,k}=\frac{N!}{N^{N}}$ and we also have that $0\leq \frac{N!}{N^{N}}\leq \frac{\left(N+1\right)!}{{\left(N+1\right)}^{N}}\leq 1$. Comment: for k=0, clearly $f_{N,k}=1$, is constant for any N. *To find the critical state probability*, $P_{c}$, *we use the fact that state probabilities add up to one*,

(23) $$\sum_{1\leq i\leq C}P_{C-i}+P_{c}+\sum_{1\leq j\leq J}P_{C+j}=1\text{.}$$

*This expression can be rewritten as*$P_{c}\left(F\left(C,\theta^{-1}\right)+1+F\left(J,\theta \right)\right)=1$*by denoting*:

(24) $$F\left(N,\alpha \right)=\underset{1\leq k\leq N}{\sum f_{N,k}}\alpha^{k}\text{.}$$

Hence $P_{c}={\left(F\left(C,\theta^{-1}\right)+1+F\left(J,\theta \right)\right)}^{-1}$. Lemma 2 *For a given value of*α>0, *the function*F(N,α)*has the following properties*:

a. F(N,α) is increasing with N
b. If α<1 then $F\left(N,\alpha \right)\leq \frac{\alpha }{1-\alpha }$.
c. For a given N, $\lim_{\alpha \to 0}F\left(N,\alpha \right)=0$.
d. For a given N, $\lim_{\alpha \to \infty }F\left(N,\alpha \right)=\infty$.

*Proof*.

a. *compare the polynomials*F(N+1,α)*and*F(N,α). *The coefficients of*$\alpha^{k}$*for*1≤k≤N*in these two functions are*$f_{N+1,k}$*and*$f_{N,k}$*respectively*. *By*Lemma 1*we know that*$f_{N+1,k}\geq f_{N,k}$. *In addition*, *the coefficient of*$\alpha^{N+1}$*in the first polynomial is*$f_{N+1,N+1}>0$*while in the second polynomial is it zero*.
b. $F\left(N,\alpha \right)=\sum_{1\leq k\leq N}f_{N,k}\alpha^{k}=\sum_{1\leq k\leq N}\frac{N!}{N^{k}\left(N-k\right)!}\alpha^{k}\leq \sum_{1\leq k\leq \infty }\alpha^{k}=\frac{\alpha }{1-\alpha }$.
c. For any 1≤k≤N, $\lim_{\alpha \to 0}\alpha^{k}=0$. $f_{N,k}$ are constants. Hence, $\lim_{\alpha \to 0}F\left(N,\alpha \right)=0$.
d. For any 1≤k≤N, $\lim_{\alpha \to \infty }\alpha^{k}=\infty$. $f_{N,k}$ are constants. Hence, $\lim_{\alpha \to \infty }F\left(N,\alpha \right)=\infty$.

*QED*. *Next*, *to find the effective arrival rate*, *we multiply each state probability by the total arrival rate in that state*.

(25) $$\lambda_{eff}=J\lambda \left(\sum_{1\leq i\leq C}P_{C-i}+P_{c}\right)+\sum_{1\leq j\leq J}P_{C+j}\lambda \left(J-j\right)$$

(26) $$\lambda_{eff}=P_{c}\lambda J\left[F\left(C,\theta^{-1}\right)+1+\sum_{1\leq j\leq J}\frac{J-j}{J}\;\right]$$

*This expression can be rewritten as*, $\lambda_{eff}=P_{c}\lambda J\left[F\left(C,\theta^{-1}\right)+1+\hat{F}\left(J,\theta \right)\;\right]$, *by denoting*:

(27) $$\hat{F}\left(N,\alpha \right)=\sum_{1\leq k\leq N}f_{N,k}\frac{N-k}{N}\alpha^{k}\text{.}$$

Lemma 3 $\hat{F}\left(N,\alpha \right)=\alpha^{-1}F\left(N,\alpha \right)-1$ and equivalently $F\left(N,\alpha \right)=\alpha \left(1+\hat{F}\left(N,\alpha \right)\right)$. *Proof*:

(28) $$F\left(N,\alpha \right)=\sum_{1\leq k\leq N}f_{N,k}\alpha^{k}=\underset{1\leq k\leq N}{\sum \;\;}\frac{N!}{N^{k}(N-k)!}\alpha^{k}$$

(29) $$F\left(N,\alpha \right)=\frac{N!}{N^{1}\left(N-1\right)!}\alpha^{1}+\sum_{2\leq k\leq N}\frac{N!}{N^{k}(N-k)!}\alpha^{k}=\alpha +\sum_{2\leq k\leq N}\frac{N!}{N^{k}(N-k)!}\alpha^{k}$$

(30) $$\hat{F}\left(N,\alpha \right)=\sum_{1\leq k\leq N}f_{N,k}\frac{N-k}{N}\alpha^{k}=\sum_{1\leq k\leq N-1}\frac{N!}{N^{k}(N-k)!}\frac{N-k}{N}\alpha^{k}$$

(31) $$\hat{F}\left(N,\alpha \right)=\alpha^{-1}\sum_{1\leq k\leq N-1}\frac{N!}{N^{k+1}(N-k-1)!}\alpha^{k+1}=\alpha^{-1}\sum_{2\leq k^{'}\leq N}\frac{N!}{N^{k'}(N-k')!}\alpha^{k'}$$

(32) $$\hat{F}\left(N,\alpha \right)=\alpha^{-1}\left(F\left(N,\alpha \right)-\alpha \right)=\alpha^{-1}F\left(N,\alpha \right)-1$$

*QED*. Lemma 4 $F\left(N,\alpha \right)-\hat{F}\left(N,\alpha \right)=1-\left(\alpha^{-1}-1\right)F\left(N,\alpha \right)$ converges to zero as α approaches zero. Proof: $\left(N,\alpha \right)-\hat{F}\left(N,\alpha \right)=\sum_{1\leq k\leq N}f_{N,k}\frac{k}{N}\alpha^{k}$, and for any 1≤k≤N, $\lim_{\alpha \to 0}\alpha^{k}=0$. *Based on*Lemma 3, *the effective arrival rate can be written as*

(33) $$\lambda_{eff}=P_{c}\lambda J\left[F\left(C,\theta^{-1}\right)+1+\hat{F}\left(J,\theta \right)\;\right]=\lambda J\frac{F\left(C,\theta^{-1}\right)+\theta^{-1}F\left(J,\theta \right)}{F\left(C,\theta^{-1}\right)+1+F\left(J,\theta \right)}\text{,}$$

(34) $$AOT=\frac{J}{\lambda_{eff\;}}=\frac{1}{\lambda }\cdot \frac{F\left(C,\theta^{-1}\right)+1+F\left(J,\theta \right)}{F\left(C,\theta^{-1}\right)+\theta^{-1}F\left(J,\theta \right)}\;\text{.}$$

(35) $$LB=\left\{ \begin{array}{cc} \frac{\theta }{\lambda } & \theta \geq 1\\ \frac{1}{\lambda } & \theta \leq 1 \end{array}\right.$$

(36) $$RID=\frac{AOT}{LB}-1=\left\{ \begin{array}{cc} \frac{AOT}{\theta /\lambda }-1=\frac{F\left(C,\theta^{-1}\right)+1+F\left(J,\theta \right)}{\theta F\left(C,\theta^{-1}\right)+F\left(J,\theta \right)}-1 & \theta \geq 1\\ \frac{AOT}{1/\lambda }-1=\frac{F\left(C,\theta^{-1}\right)+1+F\left(J,\theta \right)}{F\left(C,\theta^{-1}\right)+\theta^{-1}F\left(J,\theta \right)}-1 & \theta \leq 1 \end{array}\right.$$

(37) $$RID=\left\{ \begin{array}{cc} \frac{1-\left(\theta -1\right)F\left(C,\theta^{-1}\right)}{\theta F\left(C,\theta^{-1}\right)+F\left(J,\theta \right)} & \theta \geq 1\\ \frac{1-\left(\theta^{-1}-1\right)F\left(J,\theta \right)}{F\left(C,\theta^{-1}\right)+\theta^{-1}F\left(J,\theta \right)} & \theta \leq 1 \end{array}\right.$$

Lemma 5 pooling improves efficiency, in other words for a given load θ and servers per queue $C_{1}$, the value of RID decreases with the number of pooled queues J. *Proof*: *the total number of servers*${C=JC}_{1}$*increases with the number of pooled queues*J, *and therefore by*Lemma 2*both*F(J,θ)*and*$F\left(C,\theta^{-1}\right)$*are increasing with*J. *As a result*, *for both ranges of*θ, *the RID formula in* eq. (37) *has a decreasing numerator and an increasing denominator*. The claim in Lemma 5 about the effectiveness of pooling is qualitative. Quantitative estimates for the effect of pooling can be derived in two special cases: when comparing J=1 with J=2 with $C_{1}=1$; and when the load is balanced θ=1. In the first special case, since F(1,α)=α, and $F\left(2,\alpha \right)=\alpha +0.5\alpha^{2}$,

(38) $$RID\left(J=1,C=1,\theta \right)=\left\{ \begin{array}{cc} \frac{1-\left(\theta -1\right)F\left(1,\theta^{-1}\right)}{\theta F\left(1,\theta^{-1}\right)+F\left(1,\theta \right)}=\frac{1-\left(\theta -1\right)\theta^{-1}}{\theta \theta^{-1}+\theta }=\frac{\theta^{-1}}{1+\theta }=\frac{1}{\theta +\theta^{2}} & \theta \geq 1\\ \frac{1-\left(\theta^{-1}-1\right)F\left(1,\theta \right)}{F\left(1,\theta^{-1}\right)+\theta^{-1}F\left(1,\theta \right)}=\frac{1-\left(\theta^{-1}-1\right)\theta }{\theta^{-1}+\theta^{-1}\theta }=\frac{\theta }{\theta^{-1}+1}=\frac{1}{\theta^{-2}+\theta^{-1}} & \theta \leq 1 \end{array}\right.$$

(39) $$RID\left(J=2,C=2,\theta \right)=\left\{ \begin{array}{cc} \frac{1-\left(\theta -1\right)F\left(2,\theta^{-1}\right)}{\theta F\left(2,\theta^{-1}\right)+F\left(2,\theta \right)}=\frac{1-\left(\theta -1\right)\left(\theta^{-1}+0.5\theta^{-2}\right)}{\theta \left(\theta^{-1}+0.5\theta^{-2}\right)+\left(\theta^{1}+0.5\theta^{2}\right)}=\frac{0.5\left(\theta^{-1}+\theta^{-2}\right)}{0.5\theta^{-1}+1+\theta +0.5\theta^{2}}=\frac{1}{\theta +\theta^{2}+\theta^{3}} & \theta \geq 1\\ \frac{1-\left(\theta^{-1}-1\right)F\left(2,\theta \right)}{F\left(2,\theta^{-1}\right)+\theta^{-1}F\left(2,\theta \right)}=\frac{1-\left(\theta^{-1}-1\right)\left(\theta^{1}+0.5\theta^{2}\right)}{\left(\theta^{-1}+0.5\theta^{-2}\right)+\theta^{-1}\left(\theta^{1}+0.5\theta^{2}\right)}=\frac{0.5\left(\theta^{1}+\theta^{2}\right)}{0.5\theta^{-2}+\theta^{-1}+1+0.5\theta }=\frac{1}{\theta^{-3}+\theta^{-2}+\theta^{-1}} & \theta \leq 1 \end{array}\right.$$

*We see with or without pooling*, *RID is increasing while*θ≤1*and decreasing while*θ≥1, *with a peak at*θ=1. *The pooling improvement ratio is*:

(40) $$\frac{RID\left(J=1,C=1,\theta \right)}{RID\left(J=2,C=2,\theta \right)}=\left\{ \begin{array}{cc} \frac{\theta +\theta^{2}+\theta^{3}}{{\theta +\theta }^{2}}=1+\frac{1}{\theta^{-2}+\theta^{-1}} & \theta \geq 1\\ \frac{\theta^{-2}+\theta^{-1}+1}{\theta^{-2}+\theta^{-1}}=1+\frac{1}{\theta^{-2}+\theta^{-1}} & \theta \leq 1 \end{array}\right.$$

*The ratio is always above one*, *so pooling is indeed effective for any value of*θ, *but the improvement is monotonically decreasing*, *approaching infinity as*θ*approaches zero*, *and vanishing* (*ratio of one*) *when* θ *approaches infinity*. *To evaluate the second special case of a balanced load*, *we use an approximation for*F(N,1), *which is presented next*.

(41) $$F\left(N,1\right)=\sum_{1\leq k\leq N}f_{N,k}=\sum_{1\leq k\leq N}\frac{N!}{N^{k}(N-k)!}=\frac{N!e^{N}}{N^{N}}\sum_{0\leq i<N}\frac{N^{i}e^{-N}}{i!}$$

Now let $F_{a}\left(N\right)=\frac{N!e^{N}}{N^{N}}$, and $F_{b}\left(N\right)=\sum_{0\leq i<N}\frac{N^{i}e^{-N}}{i!}$, thus $F\left(N,1\right)=F_{a}\left(N\right)F_{b}\left(N\right)$. By the Stirling approximation $F_{a}\left(N\right)\approx {\left(2\pi N\right)}^{0.5}$. For a Poisson random variable with mean N, i.e. Y∼Pois(N), the term $F_{b}\left(N\right)=P\left(Y<N\right)$ is the probability of Y being less than its mean. By the central limit theorem, as N increases the Poisson distribution can be approximated by the normal distribution, and therefore (as is quite well known) $\lim_{K\to \infty }F_{b}\left(N\right)=0.5$. Since $P\left(Y\leq N\right)=F_{b}\left(N\right)+1/F_{a}\left(N\right)$, the sequence $F_{b}\left(N\right)+0.5/F_{a}\left(N\right)$ converges much more quickly to 0.5. Therefore, a slightly better approximation is $F_{b}\left(N\right)\approx 0.5-\frac{0.5}{F_{a}\left(N\right)}\approx 0.5-{\frac{1}{2}\left(2\pi N\right)}^{-0.5}$. A numerical evaluation of the error in this approximation suggests that an even better approximation might be $F_{b}\left(N\right){\approx 0.5-\frac{1}{3}\left(2\pi N\right)}^{-0.5}$, with a maximal absolute difference of 1E-4 in the range of 1≤N≤100. Using the latter approximation leads to the approximation $\begin{array}{c} F\left(N,1\right)\approx {\left(\frac{\pi N}{2}\right)}^{0.5}-\frac{1}{3} \end{array}$. In the range of 1≤N≤100 this approximation is slightly too low, with an error term that decreases monotonically from 0.08 to 0.01. Substituting a simpler approximation of $F\left(N,1\right)\approx {\left(\frac{\pi N}{2}\right)}^{0.5}$, in the RID for θ=1,

(42) $$RID\left(J,C=J{\cdot C}_{1},\theta =1\right)=\frac{1-\left(\theta -1\right)F\left(C,\theta^{-1}\right)}{\theta F\left(C,\theta^{-1}\right)+F\left(J,\theta \right)}=\frac{1}{F\left(C,1\right)+F\left(J,1\right)}$$

we get that:

(43) $$RID\left(J,C=J{\cdot C}_{1},\theta =1\right)\approx \frac{1}{{\left(\frac{\pi JC_{1}}{2}\right)}^{0.5}+{\left(\frac{\pi J}{2}\right)}^{0.5}}={\left(\frac{2}{\pi J}\right)}^{0.5}\frac{1}{{C_{1}}^{0.5}+1}$$

Meaning that the approximated RID is inversely proportional to the square root of the number of pooled queues. This observation explains the almost perfectly linear trends that can be observed in Fig. 7. In addition to the effect of pooling*,* the above derivations also cover the analysis of the effect of the number of servers per queue, $C_{1}$, under the assumption that the load θ remains the same*,* that is when we compare several slow servers to one fast server. In the remainder of this appendix, we examine the effect of θ *on RID*. Using eq. (37) we can show that the RID is always positive (by property b of Lemma 2). The RID converges to zero as θ approaches either zero or infinity, since in both cases the numerator converges to zero (by Lemma 4) and the denominator to infinity (by property d of Lemma 2). Lemma 6 shows that for given values of J and C, the RID peaks at θ=1, increasing monotonically up to the peak, and decreasing monotonically from there on. Lemma 6 RID is monotonically increasing when 0≤θ≤1 and monotonically decreasing when θ≥1. *Proof*: Using the notations

(44) $$\Delta \left(N,\alpha \right)=1-\left(\alpha^{-1}-1\right)F\left(N,\alpha \right)=F\left(N,\alpha \right)-\hat{F}\left(N,\alpha \right)=\sum_{1\leq k\leq N}f_{N,k}\frac{k}{N}\alpha^{k}$$

(45) $$R\left(N,M,\alpha \right)=\Delta \left(N,\alpha \right){\left[F\left(M,\alpha^{-1}\right)+\alpha^{-1}F\left(N,\alpha \right)\right]}^{-1}$$

The RID equation can be rewritten as

(46) $$RID=\left\{ \begin{array}{cc} R\left(C,J,\theta^{-1}\right) & \theta \geq 1\\ R\left(J,C,\theta \right) & \theta \leq 1 \end{array}\right.$$

To prove Lemma *6,* we need to show that R(N,M,α) is increasing with α∈[0,1] for any pair of values, N and M. The numerator in $\frac{\partial }{\partial \alpha }R\left(N,M,\alpha \right)$ can be written as the sum of three terms:

(47) $$A_{1}=F\left(M,\alpha^{-1}\right)\frac{\partial }{\partial \alpha }\Delta \left(N,\alpha \right)$$

(48) $$A_{2}=\alpha^{-2}\Delta \left(N,\alpha \right)\frac{\partial }{\partial \alpha }F\left(M,\alpha^{-1}\right)$$

(49) $$A_{3}=\alpha^{-1}F\left(N,\alpha \right)\frac{\partial }{\partial \alpha }\Delta \left(N,\alpha \right)-\alpha^{-1}\Delta \left(N,\alpha \right)\frac{\partial }{\partial \alpha }F\left(N,\alpha \right)+\alpha^{-2}F\left(N,\alpha \right)\Delta \left(N,\alpha \right)$$

We will show that all three are positive, and therefore $\frac{\partial }{\partial \alpha }R\left(N,M,\alpha \right)>0$. Both $F\left(N,\alpha \right)=\sum_{1\leq k\leq N}f_{N,k}\alpha^{k}$ and $\Delta \left(N,\alpha \right)=\sum_{1\leq k\leq N}f_{N,k}\frac{k}{N}\alpha^{k}$ are always positive and increasing with α≥0. Therefore, $\frac{\partial }{\partial \alpha }\Delta \left(N,\alpha \right)>0$ and $\frac{\partial }{\partial \alpha }F\left(M,\alpha^{-1}\right)>0$, and thus $A_{1}>0$ and $A_{2}>0$. It remains to show that $A_{3}>0$ or equivalently that $\alpha^{2}A_{3}>0$.

(50) $$\frac{\partial }{\partial \alpha }F\left(N,\alpha \right)=\sum_{1\leq k\leq N}kf_{N,k}\alpha^{k-1}=\alpha^{-1}N\cdot \Delta \left(N,\alpha \right)$$

(51) $$\frac{\partial }{\partial \alpha }\Delta \left(N,\alpha \right)=\frac{\partial }{\partial \alpha }\left[1-\left(\alpha^{-1}-1\right)F\left(N,\alpha \right)\right]=\alpha^{-2}F\left(N,\alpha \right)-\left(\alpha^{-1}-1\right)\frac{\partial }{\partial \alpha }F\left(N,\alpha \right)$$

(52) $$\frac{\partial }{\partial \alpha }\Delta \left(N,\alpha \right)=\alpha^{-2}F\left(N,\alpha \right)-\left(\alpha^{-1}-1\right)\alpha^{-1}N\cdot \Delta \left(N,\alpha \right)$$

(53) $$F\left(N,\alpha \right)\frac{\partial }{\partial \alpha }\Delta \left(N,\alpha \right)=\alpha^{-2}F^{2}\left(N,\alpha \right)-\left(\alpha^{-1}-1\right)\alpha^{-1}N\cdot F\left(N,\alpha \right)\Delta \left(N,\alpha \right)$$

Substituting

(54) $$\left(\alpha^{-1}-1\right)F\left(N,\alpha \right)\Delta \left(N,\alpha \right)=\left[1-\Delta \left(N,\alpha \right)\right]\Delta \left(N,\alpha \right)=\Delta \left(N,\alpha \right)-\Delta^{2}\left(N,\alpha \right)$$

We get the first component of $A_{3}$:

(55) $$F\left(N,\alpha \right)\frac{\partial }{\partial \alpha }\Delta \left(N,\alpha \right)=\alpha^{-2}F^{2}\left(N,\alpha \right)-\alpha^{-1}N\Delta \left(N,\alpha \right)+\alpha^{-1}N\Delta^{2}\left(N,\alpha \right)$$

*The second component of*$A_{3}$*is*:

(56) $$\Delta \left(N,\alpha \right)\frac{\partial }{\partial \alpha }F\left(N,\alpha \right)=\Delta \left(N,\alpha \right)\cdot \alpha^{-1}N\cdot \Delta \left(N,\alpha \right)=\alpha^{-1}{N\cdot \Delta }^{2}\left(N,\alpha \right)$$

And the difference between them simplifies to:

(57) $$F\left(N,\alpha \right)\frac{\partial }{\partial \alpha }\Delta \left(N,\alpha \right)-\Delta \left(N,\alpha \right)\frac{\partial }{\partial \alpha }F\left(N,\alpha \right)=\alpha^{-2}F^{2}\left(N,\alpha \right)-\alpha^{-1}N\Delta \left(N,\alpha \right)$$

Recall that:

(58) $$A_{3}=\alpha^{-1}\left[F\left(N,\alpha \right)\frac{\partial }{\partial \alpha }\Delta \left(N,\alpha \right)-\Delta \left(N,\alpha \right)\frac{\partial }{\partial \alpha }F\left(N,\alpha \right)\right]+\alpha^{-2}F\left(N,\alpha \right)\Delta \left(N,\alpha \right)$$

By substitution we get:

(59) $${\alpha^{2}A}_{3}={\alpha^{-1}F}^{2}\left(N,\alpha \right)-N\Delta \left(N,\alpha \right)+F\left(N,\alpha \right)\Delta \left(N,\alpha \right)$$

(60) $${\alpha^{2}A}_{3}=F\left(N,\alpha \right)\left[\alpha^{-1}F\left(N,\alpha \right)+\Delta \left(N,\alpha \right)\right]-N\Delta \left(N,\alpha \right)$$

Since

(61) $$\Delta \left(N,\alpha \right)=1-\alpha^{-1}F\left(N,\alpha \right)+F\left(N,\alpha \right)\;\Rightarrow \;\Delta \left(N,\alpha \right)+\alpha^{-1}F\left(N,\alpha \right)=1+F\left(N,\alpha \right)$$

We see that:

(62) $$\alpha^{2}A_{3}=F\left(N,\alpha \right)\left[1+F\left(N,\alpha \right)\right]-N\Delta \left(N,\alpha \right)$$

To show that ${\alpha^{2}D}_{3}>0$ we show that all the coefficients of $\alpha^{k}$ are non-negative, and some are positive. Recall that N⋅Δ(N,α)= $\sum_{1\leq k\leq N}kf_{N,k}\alpha^{k}$, and

(63) $$F\left(N,\alpha \right)\left[1+F\left(N,\alpha \right)\right]=\left[\sum_{1\leq k_{1}\leq N}f_{N,k_{1}}\alpha^{k_{1}}\right]\left[\sum_{0\leq k_{2}\leq N}f_{N,k_{2}}\alpha^{k_{2}}\right]\text{.}$$

Therefore*,* for N<k≤2N, the coefficient o*f* $\alpha^{k}$ is clearly positive. The coefficient of $\alpha^{k}$ for 1 ≤k≤N is:

(64) $$\sum_{1\leq k_{1}\leq k}f_{N,K_{1}}f_{N,k-k_{1}}-kf_{N,k}$$

To show that this coefficient is positive, the final step in the proof is obtained by showing that $f_{N,k_{1}}\cdot f_{N,k_{2}}\geq f_{N,k_{1}+k_{2}}$. Indeed*:*

(65) $$f_{N,k_{1}+k_{2}}=\frac{\prod_{j=0}^{k_{1}+k_{2}-1}\left(N-j\right)}{N^{k_{1}+k_{2}}}=\frac{\prod_{j=0}^{k_{1}-1}\left(N-j\right)}{N^{k_{1}}}\frac{\prod_{j=0}^{k_{2}-1}\left(N-j-k_{1}\right)}{N^{k_{2}}}\leq \frac{\prod_{j=0}^{k_{1}-1}\left(N-j\right)}{N^{k_{1}}}\frac{\prod_{j=0}^{k_{2}-1}\left(N-j\right)}{N^{k_{2}}}==f_{N,k_{1}}\cdot f_{N,k_{2}}$$

QED.
